# Small extracellular vesicles deliver TGF‐β1 and promote adriamycin resistance in breast cancer cells

**DOI:** 10.1002/1878-0261.12908

**Published:** 2021-02-24

**Authors:** Chunli Tan, Wenbo Sun, Zhi Xu, Shuyi Zhu, Weizi Hu, Xiumei Wang, Yanyan Zhang, Guangqin Zhang, Zibin Wang, Yong Xu, Jinhai Tang

**Affiliations:** ^1^ Jiangsu Institute of Cancer Research Jiangsu Cancer Hospital The Affiliated Cancer Hospital of Nanjing Medical University China; ^2^ Department of General Surgery the First Affiliated Hospital with Nanjing Medical University China; ^3^ Department of Pharmacy The People's Hospital of Guangxi Zhuang Autonomous Region Nanning China; ^4^ Jiangsu Key Lab of Cancer Biomarkers, Prevention and Treatment Nanjing Medical University China; ^5^ School of Basic Medicine and Clinical Pharmacy China Pharmaceutical University Nanjing China; ^6^ Analysis and Test Center Nanjing Medical University China

**Keywords:** adriamycin resistance and breast cancer, apoptosis, EMT, sEVs, TGF‐β1

## Abstract

Chemotherapeutic resistance is a major obstacle in the control of advanced breast cancer (BCa). We have previously shown that small extracellular vesicles (sEVs) can transmit adriamycin resistance between BCa cells. Here, we describe that sEV‐mediated TGF‐β1 intercellular transfer is involved in the drug‐resistant transmission. sEVs were isolated and characterized from both sensitive and resistant cells. sEVs derived from the resistant cells were incubated with the sensitive cells and resulted in transmitting the resistant phenotype to the recipient cells. Cytokine antibody microarray revealed that most metastasis‐associated cytokines present at the high levels in sEVs from the resistant cells compared with their levels in sEVs from the sensitive cells, particularly TGF‐β1 is enriched in sEVs from the resistant cells. The sEV‐mediated TGF‐β1 intercellular transfer led to increasing Smad2 phosphorylation and improving cell survival by suppressing apoptosis and enhancing cell mobility. Furthermore, sEV‐mediated drug‐resistant transmission by delivering TGF‐β1 was validated using a zebrafish xenograft tumor model. These results elaborated that sEV‐mediated TGF‐β1 intercellular transfer contributes to adriamycin resistance in BCa.

## Graphical abstract

Here, we show that small extracellular vesicles (sEVs) from adriamycin‐resistant breast cancer cells could confer resistance to adriamycin‐sensitive breast cancer cells. sEVs increased cell proliferation by inhibiting apoptosis and promoted cell mobility by enhancing EMT. sEV‐derived TGF‐β1 activated the TGF‐β1/Smad2 signaling pathway in recipient cells.

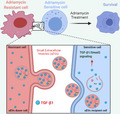

**Abbreviations**
BCa: breast cancer
ELISA: enzyme‐linked immunosorbent assay
EMT: epithelial–mesenchymal transition
HER2: human epidermal growth factor receptor 2
IL‐6: interleukin‐6
IL‐8: interleukin‐8
NF‐κB: nuclear factor kappa B
RT–qPCR: reverse transcription quantitative PCR
sEVs: small extracellular vesicles
shRNA: short hairpin RNA
Smad2/3: SMAD family member 2/3
TEM: transmission electron microscope
TGF‐β1: transforming growth factor beta 1

## 1 Introduction

Breast cancer (BCa) is the most common type of malignant cancer among women globally [1]. Owing to improved early diagnosis and advanced therapy strategies, the BCa death rates have decreased in Western developed countries, including the United States. However, distant‐organ metastasis associated with multidrug resistance and relapse still remains the main obstacle on the path to successful control of advanced BCa [2, 3]. As the first‐line proliferation inhibitor drug to treat cancers, adriamycin is widely used in postoperative adjuvant chemotherapy for BCa [4]. Although most patients have initially received the therapeutic benefits, eventually, the patients developed more aggressive tumor forms that were resistant to adriamycin. Multiple machinists have been delineated to underlie BCa adriamycin resistance, particularly NF‐κB pathway‐mediated cytokine activation is widely recognized to play a pivotal role in adriamycin resistance [5].

The drug resistance of tumor cells can be divided into natural drug resistance and acquired drug resistance. The interaction between tumor cells and microenvironment, including the production of soluble factors and vesicles, mainly contributes to the acquired drug resistance [6]. Extracellular vesicle (EV), a carrier of active molecules for intercellular communication, is thought to be related to cancer progression and drug resistance. sEVs are cell‐secreted vesicles of < 200 nm in diameter with a lipid bilayer structure [7, 8]. SEVs encapsulate intracellular components, such as miRNA and proteins [9, 10]. The molecules carried by sEVs can be transferred to surrounding cells, thereby altering phenotypes of the recipient cells [11]. Recent studies have demonstrated that sEVs are considered as important carriers of substance transport for intercellular signal transduction. For instance, sEVs conferred drug resistance to the recipient cells via transferring noncoding RNA [12, 13, 14]. In addition, P‐glycoprotein, a member of ATP‐binding cassette family, can be carried by sEVs and endow the recipient cells for docetaxel resistance [15].

Cytokines including chemokines are thought to be crucial factors in tumorigenesis, metastasis, and therapeutic resistance. Accumulating evidence has demonstrated that the treatment‐inducible cytokines contribute to therapeutic resistance in BCa [16, 17, 18]. Notably, the activation of TGF‐β, IL‐6, IL‐8, and TNF‐α apparently promotes multidrug resistance [19, 20, 21, 22]. Recent studies have found that cytokines are present at high levels in sEVs from tumor cells, predicting sEV‐mediated intercellular cytokine transfer within the tumor microenvironment. For example, under hypoxic conditions, sEVs secreted by glioma cells carry more proteins, including IL‐8 [23]. It is worth noting that stromal cell‐derived sEVs selectively transport their contents including TGF‐β to recipient tumor cells, causing changes in receptor‐related signals, thereby promoting cancer development [24, 25]. TGF‐β1 is a subtype of the TGF‐β superfamily and HER2‐targeted drug‐resistant BCa cell‐derived sEVs carry high levels of TGF‐β1, leading to enhanced resistance to anticancer immune response [26].

The present study revealed that metastasis‐relevant cytokines are enriched in sEVs from adriamycin‐resistant BCa cells, particularly TGF‐β1 uniquely presents a high level. The sEVs/TGF‐β1 was able to transfer into adriamycin‐sensitive BCa cells and enhanced the recipient cells' resistant capacity via increasing cell survival and promoting cell mesenchymal phenotype.

## 2 Materials and methods

### 2.1 Cell culture and inducing adriamycin resistance

Human breast adenocarcinoma cell line MCF‐7 was purchased from the American Type Culture Collection (ATCC, Manassas, VA, USA) and cultured in DMEM (Gibco, Gaithersburg, MD, USA) supplemented with 10% FBS (Gemini, New York, NY, USA), 100 U·mL^−1^ penicillin, and 100 µg·mL^−1^ streptomycin (Gibco) in a humidified atmosphere of 5% CO_2_ at 37 °C. Adriamycin‐resistant variant of MCF‐7 cell line was established by exposing MCF‐7 to gradually increasing concentrations of adriamycin (Pfizer, New York, NY, USA). In brief, MCF‐7 cells were treated with a series of concentrations of adriamycin to determine IC50 value. To induce the adriamycin‐resistant cell line, the cells were treated with dose‐escalated adriamycin from 0.1% IC50 to 100% IC50. After the resistant pool was stored, a single resistant cell was selected from the pool and its generation was continuously treated with increased adriamycin doses over IC50 to a final dose of 2 mm for establishing an adriamycin‐resistant cell line.

### 2.2 Cell transfection

To silence TGF‐β1, the cells were transfected with a TGF‐β1 shRNA or with a scramble control (GenePharma, Hi‐Tech Park, Shanghai, China) using a Lipofectamine reagent (Invitrogen, Waltham, MA, USA) following the manufacturer's instructions. A stable TGF‐β1‐silenced cell line was obtained using puromycin selection. MCF‐7 cells were treated with 10 ng·mL^−1^ of recombinant human TGF‐β1 (rhTGF‐β1) (PeproTech, Rocky Hill, NJ, USA) for 24 h and then removed by replacing cultural medium.

### 2.3 sEV isolation and purification

MCF‐7 cell‐secreted sEVs were isolated from cell culture media according to the update of the MISEV2018 guidelines [8]. FBS was centrifuged at 100 000 ***g***, 4 °C (Beckman, San Jose, CA, USA), for 18 h to remove serum EVs. When MCF‐7 cells were grown to 30% confluence, the medium was replaced with the EV‐free medium. The cells were continuously cultured in the EV‐free medium by 2–3 passages. Approximately 8 mL culture medium was obtained from the last subculture with 70–80% cell confluence (3 × 10^6^ adherent cells) in a 100‐mm plate (only one‐time medium drawn) for isolation of the cell‐secreted sEVs using a fractional centrifugation procedure. Briefly, 50 mL of cultural supernatants was collected from multiple plates and centrifuged at 500 ***g*** for 15 min to remove the cell debris. The supernatants were then filtered through a 0.22‐μm PVDF filter (Millipore, Hayward, CA, USA). Vesicles were precipitated by gradient centrifugation from 2000 ***g*** for 15 min, 5000 ***g*** for 15 min, to 12 000 ***g*** for 30 min. EVs were precipitated by ultracentrifugation at 100 000 ***g*** for 90 min at 4 °C. To collect sEVs, the resuspended EVs were further precipitated by repeat ultracentrifugation for 12 h. To remove contaminated proteins such as cytokines and signaling molecules, the precipitated sEVs were finally purified by passing an Amicon Ultra‐0.5 tube with a specific column for keeping EVs (Millipore).

### 2.4 Characterization of sEVs

The purified sEVs were fixed with 4% paraformaldehyde and 4% glutaraldehyde in 0.1 m phosphate buffer (pH 7.4) and then placed on a carbon‐coated copper grid and immersed in 2% phosphotungstic acid solution for examination using TEM (JEOL Ltd., Akishima, Japan) at an accelerative voltage of 80 kV. The procedure of immune electron microscope was performed to quantify TGF‐β1 protein presence in sEVs. The purified sEVs were embedded in 10% gelatin and soaked in a 2.3 m saccharose solution. sEV‐embedded gelatin ultracryotomy (70 nm) was prepared using a Leica FC7 (Leica, Vienna, Austria). The slides were incubated with 10 µg·mL^−1^ TGF‐β1 antibody (Abcam, Burlingame, CA, USA) for 1 h at 4 °C and then incubated with a goat anti‐rabbit IgG (10 nm gold) secondary antibody (Abcam) for 1 h. TGF‐β1 sorting to sEVs was examined using FEI Tecnai Spirit TEM (FEI, Santa Clara, CA, USA) at an accelerative voltage of 120 kV. To further characterize sEVs, the isolated sEVs were dissolved in a lysis buffer and analyzed by western blots with antibodies against specific EV membrane proteins.

### 2.5 sEV uptake assay

The purified sEVs were labeled with a red fluorescent dye PKH26 (Sigma‐Aldrich, St. Louis, MO, USA) according to the manufacturer's protocol. Briefly, sEVs were resuspended in a diluted buffer and incubated with diluted PKH26 for 5 min. After washing with 1 × BSA twice to remove excess dyes, the stained sEVs were recovered by ultracentrifugation. The fluorescent images were analyzed using a fluorescence microscope (Nikon, Tokyo, Japan). To label the cells, 3 × 10^5^ MCF‐7 cells were seeded in 6‐well plates overnight and then infected with a lentivirus encoding GFP (GenePharma, Hi‐Tech Park, Shanghai, China). The cells were cultured for 3 days to allow GFP expression observed under the fluorescence microscope. A stable GFP‐labeled cell line was obtained by puromycin selection. The GFP‐labeled cells were grown on a confocal dish with EV‐free medium and maintained to 25% confluence. The cells were incubated with PKH26‐labeled sEVs for 24 h and then fixed in 4% paraformaldehyde for 15 min. After washing with PBS, the cells were permeabilized with ice‐cold 0.5% Triton X‐100 for 20 min at room temperature. The samples were subjected to probing with the appropriate primary and secondary antibodies. The nuclei were counterstained with DAPI (Invitrogen, P‐36931) according to the manufacturer's instructions. The fluorescence was visualized and captured with confocal microscopy (Leica, Wetzlar, Germany).

### 2.6 Co‐localization of TGF‐β1 with sEVs in the recipient cells

After cell‐sEV incubation, uptake of sEVs was imaged using a CD63 antibody with red fluorescence (Santa Cruz Biotechnology, Santa Cruz, CA, USA), and TGF‐β1 was imaged using a TGF‐β1 antibody with green fluorescence (Abcam). In addition, to control the co‐localization of TGF‐β1 with CD63, ER‐associated calnexin protein and nuclei‐associated LamB1 protein were imaged using their relative antibodies with red fluorescence (Proteintech, Rosemont, IL, USA). The fluorescence was visualized and captured with confocal microscopy (Leica).

### 2.7 Cell survival analysis

MCF‐7 cells were cultured in 6‐well plates with EV‐free medium. When cell density reached 30% confluence, sEVs isolated from 50 mL of cultural supernatants were added to each well and continuously cultured for 3 days. Adriamycin‐mediated cytotoxicity was analyzed by MTT assay and clonogenic assay. For MTT assay, the cells were plated into 96‐well plates at 10^3^ cells per well. Twelve hours after preculture, the medium was replaced by media containing a series of concentrations of adriamycin at 0.125, 0.25, 0.5, 1, 2, and 4 μm. Forty‐eight hour after treatment, MTT reagent was applied according to manufacturer's instruction and cell viability was determined using a microplate reader (Thermo Multiskan, Waltham, MA, USA) at 562 nm. For clonogenic assay, the cells were seeded into 6‐well plates at 200 cells/well and maintained for 24 h. The cells were treated with 0, 3, 6, 8, 10, 20, and 30 ng·mL^−1^ of adriamycin for 21 days to allow colony formation. The colonies were stained with 1% crystal violet and then counted.

### 2.8 Apoptosis assay

Flow cytometry analysis was used to quantify apoptotic cells after treatment. 1 × 10^5^ cells were seeded in 6‐well plates overnight. After washed with PBS, the cells were incubated with S/sEVs or A/sEVs in FBS‐free media for 3 days. sEVs were dissolved by replacing media and then treated with 10 μm adriamycin for 24 h to induce apoptosis. Both of the attached and floating cells were harvested, and flow cytometry analysis was performed using an Annexin V‐FITC/PI Staining Kit (Dojindo Molecular Technologies, Kumamoto, Japan) according to the manufacturer's instructions. Apoptotic cells were quantified under the detection of a BD FACSCalibur Flow Cytometer (BD Sciences, San Jose, CA, USA).

### 2.9 Wound‐healing assay

3 × 10^5^ cells in serum‐free DMEM on six‐well plates were rinsed three times with PBS, and then, a sterile 200‐μL pipette tip was used to create three separate parallel wounds. After washing several times with PBS, floating cells were removed. The cells were continuatively cultured in the completed media. The wound closure was monitored after 24 h using a microscope. Three independent experiments were performed. The wound surface area was quantified using ImageJ, NIH (Bethesda, MD, USA).

### 2.10 Cell motility

Cell migration and invasion assays were performed in 24‐well transwell chambers with 8‐μm‐pore polycarbonate filter inserts (Corning Inc., Sunnyvale, CA, USA). The cells were seeded at a density of 2 × 10^5^ in uncoated or Matrigel‐coated (BD Bioscience, San Jose, CA, USA) inserts with 200 μL serum‐free media, respectively. The lower chambers were filled with 500 μL of 10% FBS‐supplemented DMEM as chemoattractant. After 24 h, cells on the upper side of the filter were removed and those on the lower surface of the insert were stained with crystal violet (Beyotime, Beijing, China) for 20 min and counted using a microscope.

### 2.11 Human cytokine antibody array

Human cytokine antibody array was performed to analyze sEV cytokine profiling by RayBiotech, Nanjing, China, according to the manufacturer's instructions. Briefly, 50 µg sEV sample was used to extract proteins and subjected to the array. After the reaction was blocked, followed by incubation with biotin‐conjugated antibodies for 2 h and then with HRP‐linked secondary antibody for 1 h. The membranes were incubated with chemiluminescent substrate and exposed to X‐ray film for 15 min. Array Vision Evaluation 8.0 (GE Healthcare, Sunnyvale, CA, USA) was used for quantitative analysis.

### 2.12 Western blot analysis

Whole‐cell and sEV lysates were prepared by adding RIPA lysis buffer containing 1 mm PMSF, and protein concentration was measured using a BCA Assay Kit (KeyGen Biotech, Nanjing, China). 10–20 µg extracted proteins were separated on gradient SDS/PAGE gels and then transferred to PVDF membranes. The membranes were subsequently incubated at 4 °C overnight with the primary antibodies, including Calexin, Alix, TSG101, CD63, TGF‐β1, GAPDH, Smad2, phopho‐Smad2, E‐cadherin, Snail, Twist 1, and β‐actin. Thereafter, the membranes were incubated at room temperature for 2 h with HRP‐conjugated secondary antibody (Santa Cruz Biotechnology). All blots were detected using the enhanced chemiluminescence (ECL) with ChemiDoc™ XRS+ imaging system (Bio‐Rad, Irvine, CA, USA). quantity one software (Bio‐Rad) was applied to evaluate the image of blots and further normalized by GAPDH and β‐actin.

### 2.13 ELISA

The levels of TGF‐β1 were measured using the Quantikine Human TGF‐β1 Immunoassay Kit (R&D Systems Inc., Minneapolis, MN, USA) according to the manufacturer's instructions. The cell lysates were added into a 96‐well plate coated with a monoclonal antibody against TGF‐β1. After incubation, a substrate solution for the immunoperoxidase reaction was added to develop a color based on the amount of TGF‐β1 antigen present in the samples. The reaction was terminated by adding a stop solution. TGF‐β1 concentration was quantitated by measuring the absorbance of each well at 450 nm based on the standard curve.

In addition, the Smad2 ELISA Kit (ABclonal, Woburn, MA, USA) was used to determine the nuclear translocation of Smad2 in the recipient cells. After cell‐sEV incubation, the nuclear fraction and cytoplasmic fraction were separated and extracted from 2 × 10^7^ recipient cells using a Nuclear and Cytoplasmic Protein Extraction Kit (Beyotime Biotechnology) according to the manufacturer's instruction. Nuclear proteins and corresponding cytoplasmic proteins extracted from the same amount of cells were subjected to the ELISA Kit to quantify Smad2. The percentage of nuclear translocated Smad2 was calculated by dividing with total Smad2.

### 2.14 RNA isolation and RT–qPCR

Total RNA was extracted from the treated cells by TRIzol reagent and qualified by agarose gel electrophoresis. 1.5 µg RNA was converted to cDNA using a Mir‐X miRNA First‐Strand Synthesis Kit (Takara, Tokyo, Japan). Twenty nanogram cDNA was used to perform RT**–**qPCR was performed using a SYBR Premix Ex Taq II Kit (Takara) and detected by a LightCycler System (Roche, Santa Clara, CA, USA). The relative expression level of TGF‐β1 mRNA was quantified by normalizing with GAPDH. Sequences of the specific PCR primers for TGF‐β1 are forward primer, 5′‐TATCGACATGGAGCTGGTGA‐3′; reverse primer, 5′‐GTGGGTTTCCACCATTAGCA‐3′; and that for GAPDH are forward primer, 5′‐TCTGACTTCAACAGCGACACC‐3′, reverse primer, 5′‐CTGTTGCTGTAGCCAAATTCGTT‐3′.

### 2.15 *In vivo* study using a zebrafish model

A zebrafish tumor experimental model was established for *in vivo* study. The ethical approval for the tumor zebrafish xenograft experiment is included in the Institutional Animal Care and Use Committee approved by the Research Committee of Nanjing Medical University (No. IACUC‐1901031). MCF‐7 cells were incubated with S/sEVs and A/sEVs in BSF‐free media for 3 days. After washing with 1× BPS, the cells were incubated with BSF‐free media containing PKH26 fluorescent dye at 37 °C for 20 min. 200 PKH26‐labeled cells were microinjected into a zebrafish embryo (aged at 48 hpf). The injected cells were attached to the yolk and evenly spread throughout the organism. Each experimental group contained more than 20 successfully injected embryos and 10–15 fishes. The injected embryos were incubated at 28 °C for 1 h and then turned to 34 °C to continue the cultivation. Twenty‐four hours after cell injection, 50 µm adriamycin was added into zebrafish nutrient solution to inhibit tumor growth. 7–10 days after treatment, the tumors formed in adult fishes were imaged on a fluorescent microscope at 583 nm and quantified using an imagej software (NIH).

### 2.16 Statistical analysis

All experiments have been repeated multiple times, the quantitative analyses were performed with at least three replicates, and the data were presented as the mean ± standard deviation. Unpaired two‐tailed Student's *t*‐test was used to analyze the significance between two groups. For multiple comparisons, one‐way analysis of variance (ANOVA) followed by Dunnett's or Bonferroni's test was performed using graphpad prism (GraphPad, San Diego, CA, USA). *P* < 0.05 was considered statistically significant.

## 3 Results

### 3.1 Isolation and characterization of sEVs

MCF‐7 cells were consistently treated with dose‐escalated adriamycin, and a resistant cell line (MCF‐7/Adr) was selected and characterized. The results of colony survival assay verified that MCF‐7/Adr cells appeared to be highly resistant to adriamycin as compared to the parental MCF‐7 cells (Fig. S1A,B). Consistently, MTT assay also showed that the survival rate of MCF‐7/Adr cells was significantly higher than that of MCF‐7 cells after adriamycin treatment. The mean IC50 of adriamycin was 101.7 μm in MCF‐7/Adr cells, but only 2.3 μm in MCF‐7 cells (Fig. S1C).

**Fig. 1:**
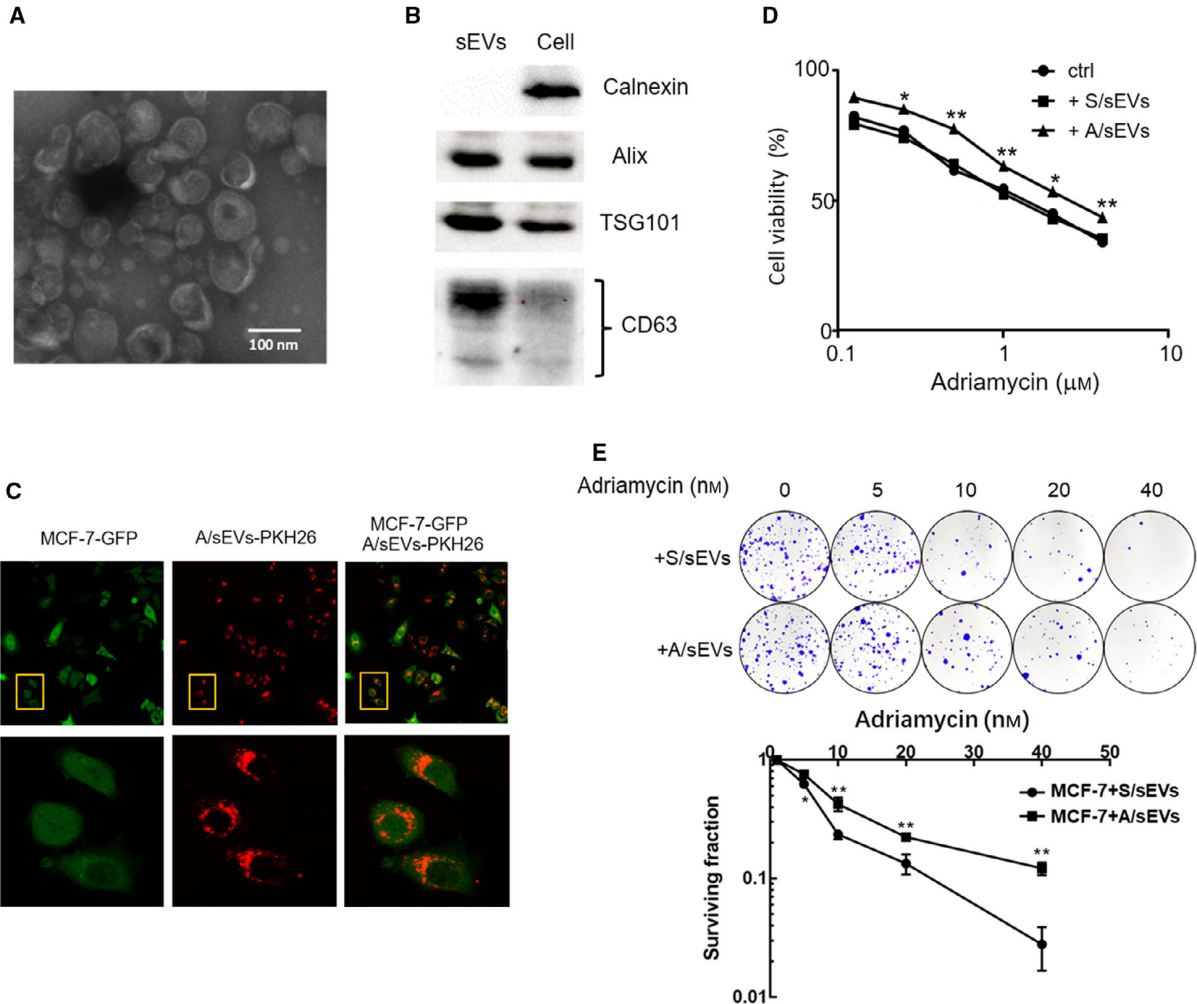
sEV‐mediated intercellular transmission of adriamycin resistance. (A) sEVs were isolated from the culture medium of MCF/Adr cells and characterized by TEM. (B) The isolated sEVs were further examined by the determination of exosome‐specific proteins CD63, TSG101, and Alix by western blots. Calnexin serves as a cytosolic protein control. (C) MCF‐7 cells were labeled with GFP, and A/sEVs were labeled with PKH26 (red). After incubation of cells with sEVs, sEV intracellular localization was observed using a confocal microscope. Images in the low panel were representative for images in boxes as indicated in the top panel. (D) MCF‐7 cells were incubated with S/sEVs or A/sEVs and then treated with different concentrations of adriamycin as indicated. The cell viability after normalized with cell plating efficiency was analyzed by MTT assay. No sEVs added were a negative control. (E) After cell‐sEV incubation and drug treatment, adriamycin resistance was analyzed by cell colony survival assay. All experiments were repeated, and more than three replicates were included in D and E. The data were presented as the mean ± standard deviation. *P < 0.05 and **P < 0.01 represent the significances between two groups as indicated.

sEVs were isolated from cell cultural supernatants using ultracentrifugation, whose morphological characteristics were examined under TEM. The sEVs exhibited lipid bilayer membrane structures, diameters of < 200 nm in size (Fig. 1A). In addition, the isolated sEVs were further characterized by western blots using specific antibodies against multivesicular proteins, such as TSG101 and CD63 and Alix. Their levels in sEVs were apparently higher than the levels in whole‐cell extracts, although the same protein amounts were loaded. In contrast, calnexin, a cytoplasmic protein, served as a negative control that was only detected in cytosol fractions (Fig. 1B).

### 3.2 Adriamycin‐resistant cell‐derived sEVs enhance resistance of the sensitive recipient cells

To examine whether MCF‐7/Adr cell‐derived sEVs (A/sEVs) can transfer into the parental MCF‐7 cells, A/sEVs were labeled with PKH26 red fluorescence and MCF‐7 cells were labeled with GFP, respectively. After incubation of cells with A/sEVs, as expected, the labeled sEVs can be detected in the recipient cells, which are mainly localized in the cytoplasm (Fig. 1C). To testify whether MCF‐7/Adr cell‐derived sEVs can transmit drug resistance to MCF‐7 cells, after cell‐sEV incubation, the cells were treated with different doses of adriamycin. Treatment‐induced cytotoxicity was analyzed by MTT assay. The results showed that cell viabilities were increased in the cells incubated with A/sEVs compared with MCF‐7 cell own sEVs (S/sEVs) or no sEV uptake control (Fig. 1D). Furthermore, cell colony survival assay was conducted to verify whether A/sEVs can transmit the drug‐resistant phenotype to the recipient cells. Consistently, the drug resistance of MCF‐7 cells was strikingly enhanced after incubation with A/sEVs compared to incubation with S/sEVs (Fig. 1E). These results suggest that the drug resistance can be transmitted between cells via sEV delivery.

### 3.3 TGF‐β1 is enriched in adriamycin‐resistant cell‐derived sEVs

**Fig. 2:**
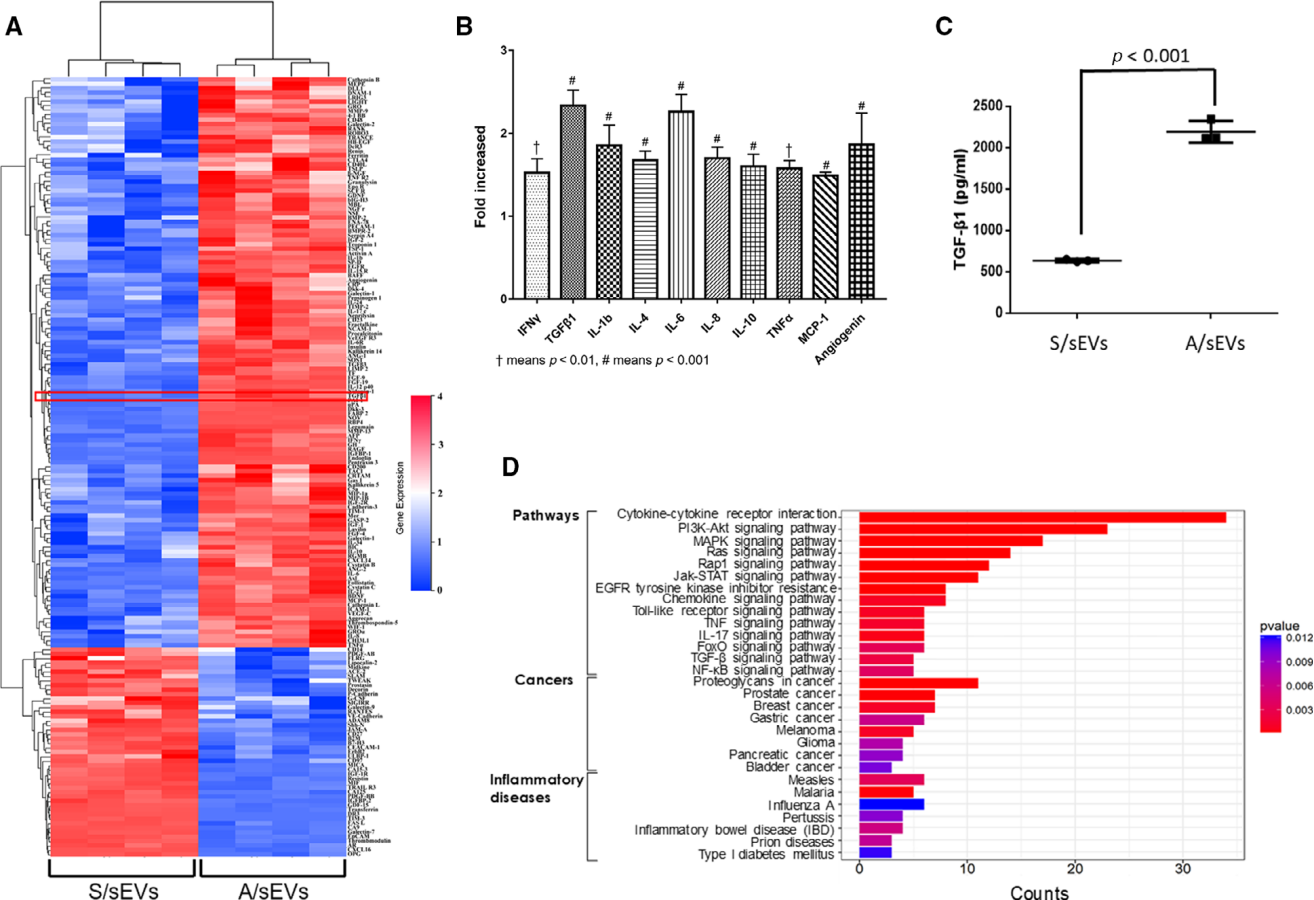
Cytokine abundances in A/sEVs vs. S/sEVs. (A) sEVs isolated from MCF‐7 and MCF‐7/Adr cells were subjected to a human cytokine antibody array to quantify the levels of targeted cytokines. A red box indicated TGF‐β1 profiling. (B) Increased abundances of BCa metastasis‐associated cytokines in A/sEVs vs. S/sEVs. (C) The amounts of TGF‐β1 in A/sEVs and S/sEVs were quantified using a specific ELISA Kit. (D) Alters in cell signaling pathways and relating inflammatory disease and cancers were analyzed using a KEGG enrichment analysis software. Each group of sEVs containing four replicates were quantified, and the significant differences between S/sEVs and A/sEVs were indicated in B and C.

In addition to DNA and RNA fragments, peptides and proteins were also detected in sEVs, such as heat‐shock proteins and cytokines [27, 28]. Since cytokines are widely recognized as potent factors for the acquisition of multidrug resistance in BCa [29], the cytokine profiling in A/sEVs vs. S/sEVs was quantified using a human cytokine antibody array. A majority of cytokines were increased in A/sEVs compared with S/sEVs (Fig. 2A). Notably, several BCa metastasis and therapeutic resistance‐associated cytokines were presented at high levels in A/sEVs (Fig. 2B). In particular, the results from ELISA further confirmed that TGF‐β1 is highly enriched in A/sEVs (Fig. 2C). Furthermore, KEGG pathway analysis revealed that the ratios of cytokines between A/sEVs and S/sEVs apparently activate multiple oncogenic pathways, such as TGF‐β, Toll, Jak‐STAT, and MARK, which consequently promote metastasis of several types of cancer through enhancement of EMT and immune evasion (Fig. 2D and Fig. S2A–E). TGF‐β1 is thought to contribute toward BCa adriamycin resistance [30]. Accordingly, the abundance of TGF‐β1 in MCF‐7/Adr cells was much higher than the level in MCF‐7 cells, suggesting that the abundances of cytokines in sEVs are mainly dependent on their expression levels in the corresponding cells (Fig. S3A,B).

### 3.4 sEVs/TGF‐β1 contributes to the drug resistance of the recipient cells

**Fig. 3:**
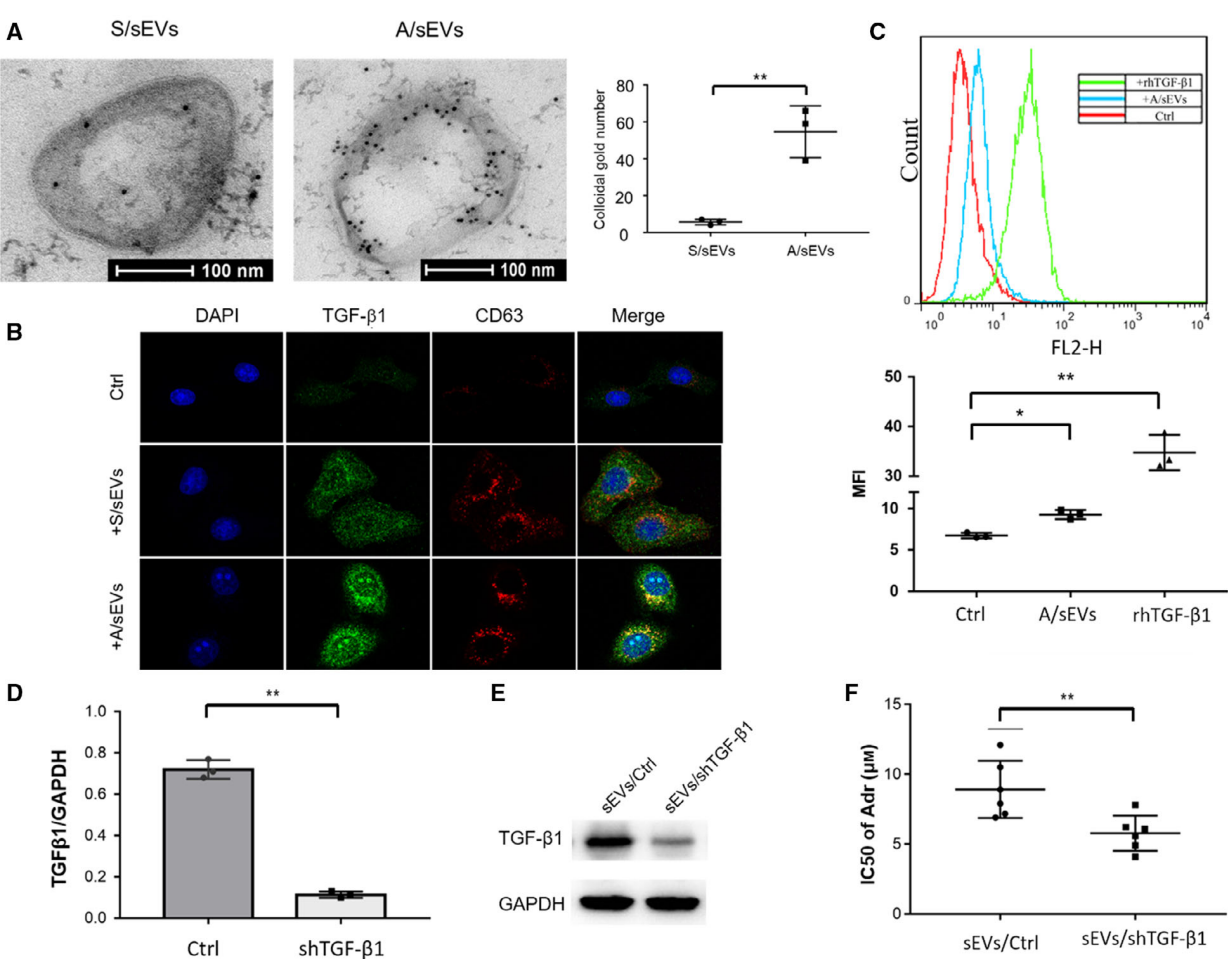
The effect of sEVs/ TGF‐β1 on intercellular adriamycin‐resistant transmission. (A) TGF‐β1 sorting to sEVs was determined by an immune electron microscope. sEV‐embedded gelatin ultracryotomy was incubated with TGF‐β1 antibody for staining sEV‐carried TGF‐β1, and the images were observed by TEM. The amounts of TGF‐β1 sorting to sEVs were quantified. (B) After cell‐sEV incubation, intracellular co‐localization of TGF‐β1 with uptake of sEVs was verified by confocal microscope using TGF‐β1 antibody (green) and CD63 antibody (red). (C) TGF‐β1 abundance was quantified using flow cytometry with the TGF‐β1 antibody. (D) TGF‐β1 in MCF‐7/Adr cells was knocked down using a specific shRNA and confirmed by RT–qPCR. A scramble sequence serves as a control. (E) sEVs were isolated from the TGF‐β1‐silenced MCF‐7/Adr cells, and the reduced level of TGF‐β1 was confirmed by western blots. (F) MCF‐7 cells were incubated with the isolated sEVs and then treated with adriamycin. The cytotoxicity was quantified by MTT assay and calculated IC50 value. All experiments were repeated, and more than three replicates were included in A and C–F. The data were presented as the mean ± standard deviation. *P < 0.05 and **P < 0.01 represent the significances between two groups as indicated.

TGF‐β1 is thought to be a key factor in BCa metastasis and therapeutic resistance [31]. To determine that sEVs can carry and enrich TGF‐β1, the purified sEVs from the sensitive and resistant cells were incubated with TGF‐β1 antibody and then imaged under TEM. As shown in Fig. 3A, TGF‐β1 appeared sorting to membrane of sEVs, particularly it was highly enriched in A/sEVs than S/sEVs. To testify whether A/sEVs can transfer TGF‐β1 to recipient cells, after cell‐sEV incubation, the internalized sEVs/TGF‐β1 was determined in the recipient cells (Fig. 3B). Importantly, TGF‐β1 was mainly overlapped with EV membrane protein CD63, but not with endoplasmic reticulum membrane protein calnexin or nuclear envelope marker Lamin B1 (Fig. S4). The observation was further confirmed by flow cytometry using a TGF‐β1 antibody (Fig. 3C). Furthermore, to verify whether sEVs/TGF‐β1 confers the drug resistance to the recipient cells, TGF‐β1 was silenced in MCF‐7/Adr cells using a TGF‐β1 shRNA (Fig. 3D,E). Accordingly, the drug resistance was alleviated in the recipient cell uptake of TGF‐β1‐silenced sEVs compared with the scrambled control (Fig. 3F).

### 3.5 sEVs/TGF‐β1 inhibits apoptosis of the recipient cells

**Fig. 4:**
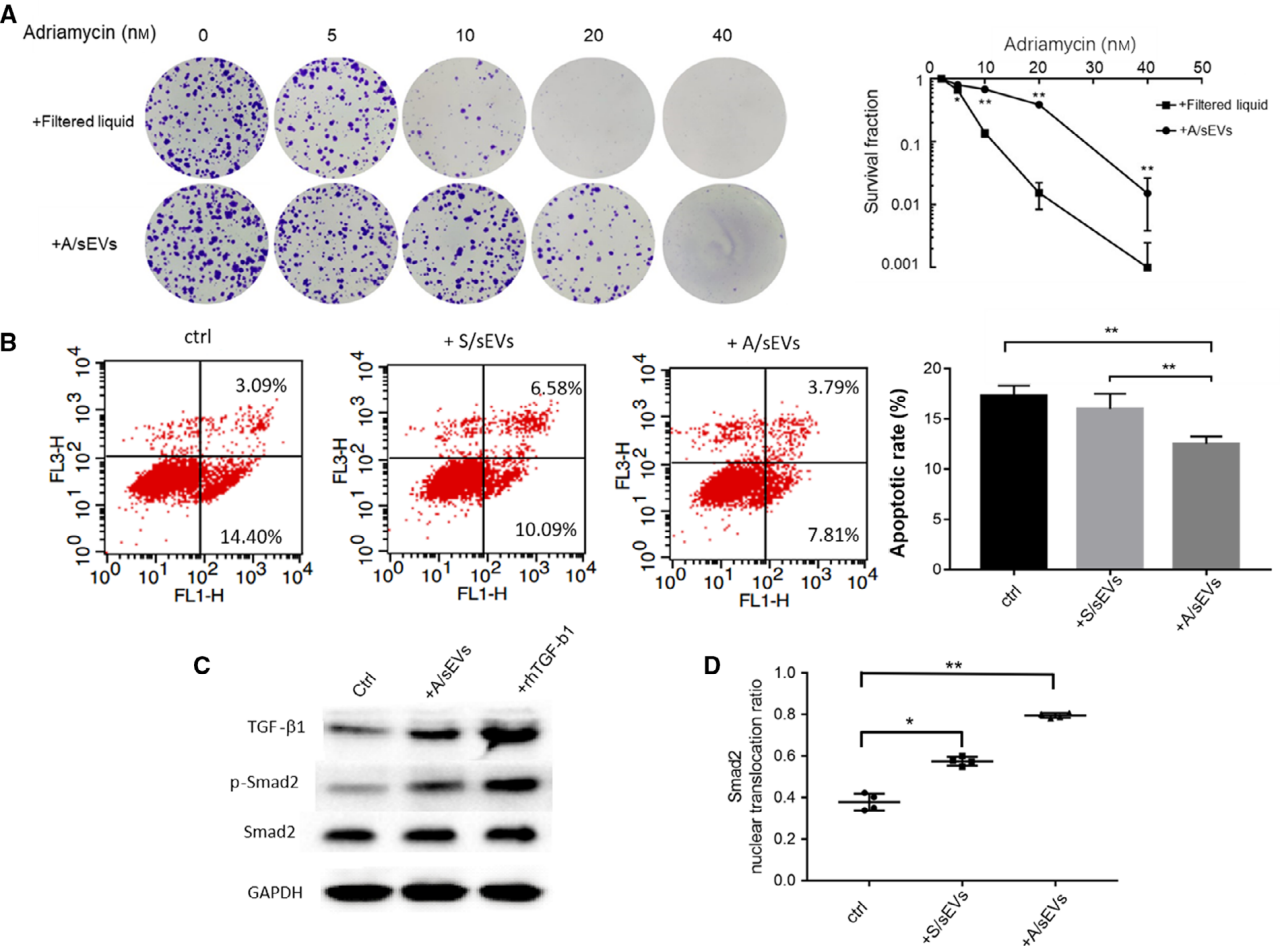
The effects of sEVs on inhibition of apoptosis in the recipient cells. (A) The isolated A/sEVs were finally purified using a specific column to remove soluble molecules accompanied by sEVs, and the eluted sEVs and filtered liquids were incubated with MCF‐7 cells. After treated with adriamycin as indicated, the cell survival rate was determined by clonogenic assay. (B) MCF‐7 cells were incubated with S/sEVs and A/sEVs and then treated with adriamycin, and the apoptotic cells were quantified by flow cytometry. The reduction in apoptosis by uptake of sEVs was calculated. **P < 0.01 represents the significances between the two groups as indicated. (C) The levels of TGF‐β1 and relating phosphorylated Smad2 in the recipient cells were quantified by western blots. Treatment with rhTGF‐β1 protein was used as a positive control. (D) Nuclear and cytoplasmic Smad2 proteins in the sEV uptake cells were measured using a Smad2 ELISA Kit. The percentage of nuclear‐translocated Smad2 was calculated by normalized with total Smad2. All experiments were repeated, and more than 3 replicates were included in A, B, and D. The data were presented as the mean ± standard deviation. *P < 0.05 and **P < 0.01 represent the significances between two groups as indicated.

It is important to confirm the contribution of sEV‐mediated molecular transferring to adriamycin resistance, but not because of molecular contamination that occurred during isolation of sEVs. The isolated A/sEVs were subjected to a specific column for removing the potentially contaminated molecules accompanied by the sEV preparation. The column‐passed fraction and column‐elution fraction were separately incubated with the sensitive cells. As shown in Fig. 4A, the eluted A/sEVs prominently increased cell survival rate compared with the filtered liquid. It has been widely recognized that the induction of apoptosis is a main biological basis for adriamycin‐mediated cell death [32]. Accordingly, the cell apoptotic rate was quantified by flow cytometry after cell‐sEV incubation and adriamycin treatment, and preapoptotic and apoptotic cell numbers were significantly reduced in cells incubated with A/sEVs compared with the use of S/sEVs (Fig. 4B).

Moreover, it has been well documented that the activation of TGF‐β classical pathway increases Smad2 phosphorylation and leads to transcriptional activation of downstream presurviving genes [33]. After cell‐sEV incubation, the level of Smad2 phosphorylation in the recipient cells was quantified. Consistent with increasing TGF‐β1, the levels of total Smad2 and phosphorylated Smad2 increased in the uptake of A/sEV cells, corresponding to results from cells treated with rhTGF‐β1 protein (Fig. 4C). Additionally, nuclear and cytoplasmic Smad2 levels were measured. The result showed that Smad2 was efficiently translocated into the nuclei in the cell uptake of A/sEVs compared with uptake of S/sEVs (Fig. 4D). The results suggest that sEV‐transported TGF‐β1 was activated in the recipient cells.

### 3.6 sEVs/TGF‐β1 promotes EMT of the recipient cells

**Fig. 5:**
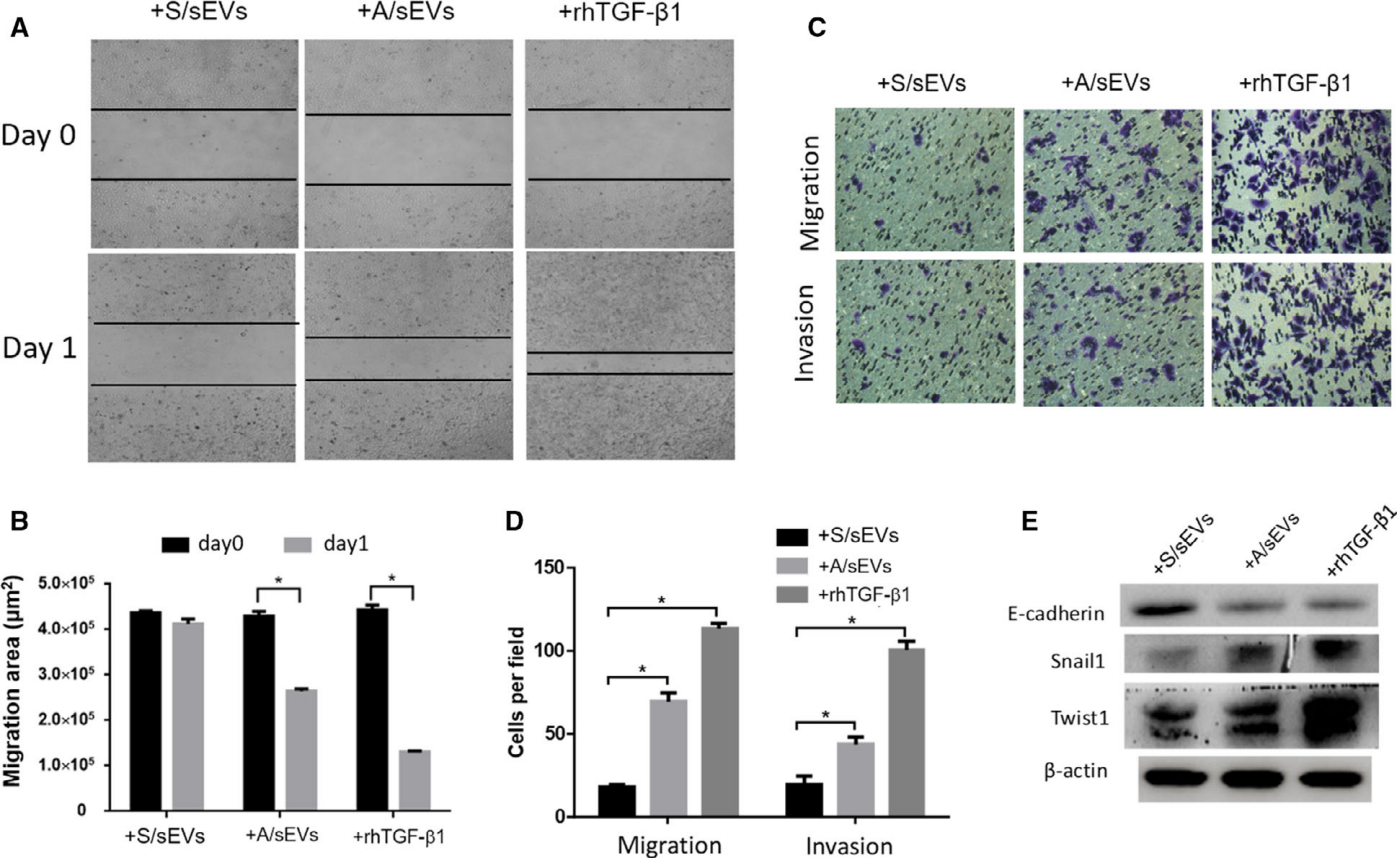
The effects of sEVs on EMT activation in the recipient cells. (A) After cell‐sEV incubation, the ability of wound closure of the recipient cells was measured using cell wound‐healing assay. (B) 24 h after incubation, unclosed areas were calculated. (C) After cell‐sEV incubation, cell invasion and migration capacities were analyzed by transwell assay. (D) The numbers of invaded and migrated cells were plotted. (E) The EMT markers expressed in the recipient cells were measured by western blots. Treatment of rhTGF‐β1 was used as a positive control. All experiments were repeated, and more than three replicates were included in A–D. The data were presented as the mean ± standard deviation. *P < 0.05 represents the significances between two groups as indicated.

TGF‐β1 is not merely the elimination of treatment‐induced cytotoxicity, but more importantly, it can activate EMT for promoting drug resistance [33]. To examine whether sEVs/TGF‐β1 enhances the EMT process in the recipient cells, after cell‐sEV incubation, several EMT phenotypic parameters were examined. Twenty‐four hours after sEV uptake, wound healing was obviously improved in A/sEV uptake cells compared with S/sEV uptake cells as a negative control or rhTGF‐β1‐treated cells as a positive control (Fig. 5A,B). Consistently, 3 days after sEV uptake, cell invasion and migration capacities were highly increased (Fig. 5C,D). In addition, the levels of several EMT relative proteins were further quantified. Accordingly, E‐cadherin was decreased, but Snal1 and Twist1 were increased in cells incubated with A/sEVs or treated with rhTGF‐β1 compared to the cells incubated with S/sEVs (Fig. 5E).

### 3.7 sEV‐mediated adriamycin‐resistant transmission is validated in zebrafish

**Fig. 6:**
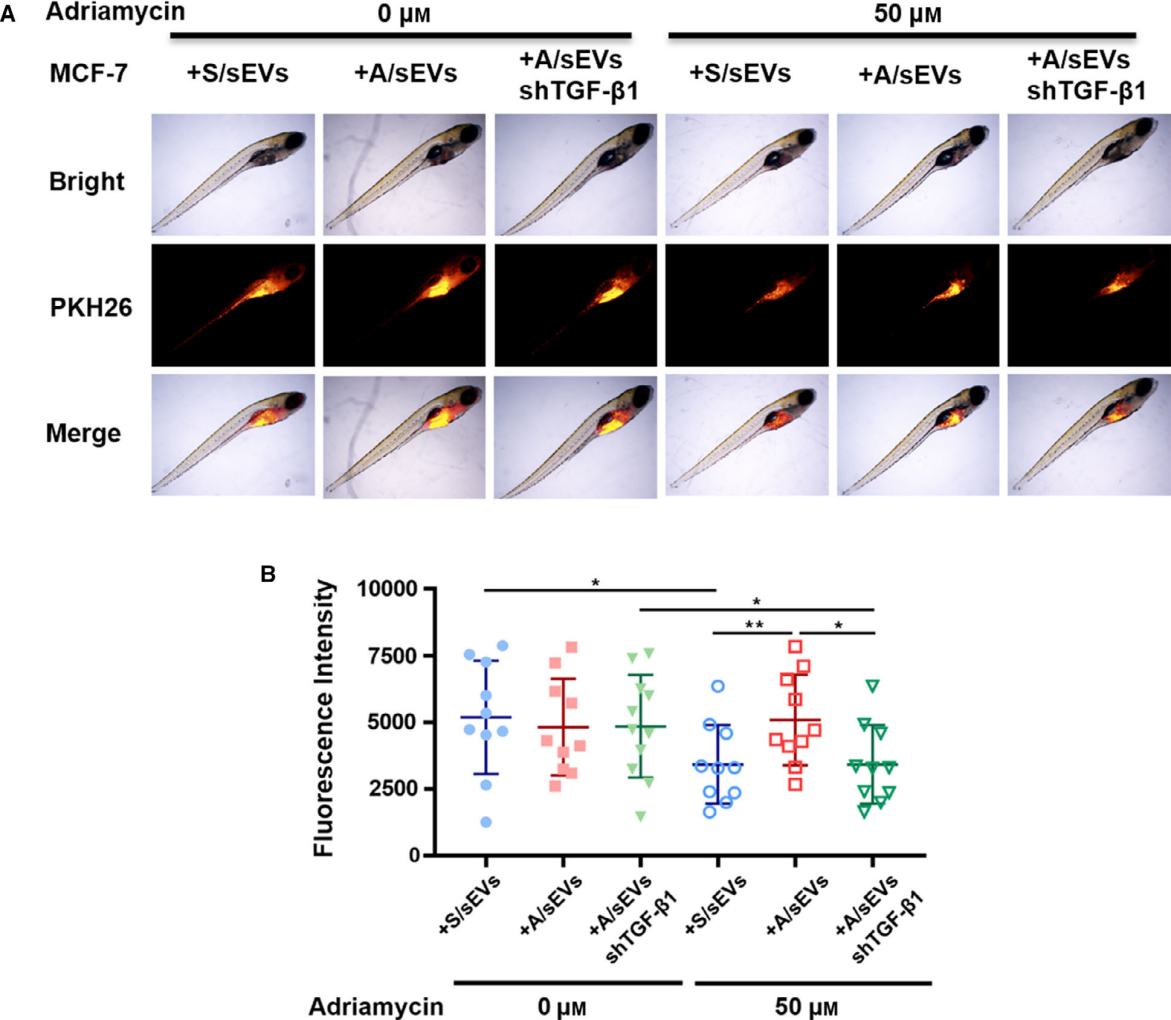
Exosome‐mediated drug resistance transmission in MCF‐7 cell‐formed tumors in zebrafish. (A) PKH26‐labeled MCF‐7 cells were incubated with S/sEVs, A/sEVs, and TGF‐β1 silenced A/sEVs. The exosome‐internalized cells were injected into zebrafish embryos. The hatched fishes were grown with 50 µm adriamycin or left the drug as a negative control. The formed tumors in the fish bodies were screened as indicated. (B) The tumor volumes were calculated and plotted. 10–15 fishes were grown in each experimental group, and the data were presented as the mean ± standard deviation. *P < 0.05 and **P < 0.01 represent the significances between two groups as indicated.

A zebrafish tumor experimental model was established to validate the above results *in vivo*. MCF‐7/Adr cells and MCF‐7 cells were injected into zebrafish embryos and then treated with adriamycin until the fishes were born. Tumors were formed as the fish grown‐up and screened by fluorescent imaging. The results showed that adriamycin treatment efficiently inhibited tumor growth, but its anticancer efficacy was significantly reduced in the group injected with MCF‐7/Adr cells (Fig. S4A,B). To further validate that A/sEVs transmit the drug resistance to the MCF‐7 cells mainly through transferring TGF‐β1, MCF‐7 cells were incubated with S/sEVs and A/sEVs. The sEV‐internalized cells were further injected into the zebrafish embryos. Intriguingly, in comparison with the uptake of S/sEV group, the drug resistance was enhanced in the uptake of A/sEV group, but the resistance was eliminated in the use of A/sEVs isolated from the TGF‐β1‐silenced resistant cells (Fig. 6A,B).

## 4 Discussion

Although the mortality rate of BCa has been continuously decreasing, distant‐organ metastasis and multidrug resistance‐related malignant recurrence remain the significant obstacles in the successful control of the disease [3]. Numerous factors and multiple mechanisms underlying chemoresistance have been uncovered. In particular, as critical inflammatory factors, cytokines/chemokines are highly related to BCa chemoresistance [34]. Notably, cytokine‐enhanced EMT is thought to play a pivotal role in the progression of metastatic phenotypes that are resistant to chemotherapy [35, 36]. Although the cytokine network is dramatically complicated, we have recently concluded that TGF‐β, IL‐6, IL‐8, and TNF‐α as major regulatory cytokines mainly contribute to BCa chemoresistance [37]. The present study further demonstrated that sEV‐delivered TGF‐β1 implicates in the intercellular transmission of adriamycin resistance in BCa cells.

TGF‐β1, a multifunctional cytokine in TGF‐β family, is uniquely elevated in the plasma of BCa patients and contributes to metastasis and therapeutic resistance [38]. Upregulation of TGF‐β1 led to abnormal activation of the TGF‐β signaling pathway in BCa cells and enhanced cell proliferation and multidrug resistance via phosphorylation of Smad2/3 [31, 39, 40]. In addition, TGF‐β1 can induce EMT and promote BCa cell migration through Smad‐dependent and Smad‐independent pathways [41]. The present study used a human cytokine antibody array to screen sEVs isolated from adriamycin‐sensitive and adriamycin‐resistant BCa cells. Most cytokines were detected in sEVs, particularly abundances of chemotherapy‐responsive cytokines that are correlated with adriamycin resistance, including TGF‐β1, IL‐6, and IL‐8. Uptake of sEV‐delivered cytokines contributed to the intercellular transmission of drug resistance, but the effects were further attenuated when TGF‐β1 was silenced.

sEVs are nanoscale vesicles secreted by various types of cells, containing a variety of intracellular active components of the parental cells, which can be transferred to surrounding cells, regulating cell survival, proliferation, invasion, and response to treatment [11]. In addition to DNA and RNA, EVs are able to carry other molecules including proteins and fatty acids. Emerging evidence that EVs deliver soluble factors such as cytokines has brought extensive attention to the investigation of the substantial roles of EV‐delivered cytokines in cancer progression and therapeutic resistance [26, 36]. In this regard, the present study used a simple sEV uptake cell model to monitor that sEV‐mediated cytokine intercellular transfer contributes to adriamycin resistance of BCa cells. Indeed, the efficiency of drug‐resistant transmission by sEVs was empowered compared with merely co‐culture of the sensitive cells with the resistant cells (data not shown), suggesting that the drug resistance‐related molecules could be concentrated in sEVs. Overall, EV‐mediated signal transaction is generally referred bystander effects, which has been emphasized in other sets of investigation [42, 43].

EV‐mediated cytokine transfer within the tumor microenvironment appears to be a major concern in metastasis and relating drug resistance. sEVs have been delineated as vehicles to transfer IL‐6/STAT3 into recipient cells, which subsequently activated several oncogenic pathways [24, 44]. TNF and TNFR have been detected in sEVs of melanoma cells and were able to transmit redox signaling to adjacent cells, leading to tumor immune escape [45]. SEVs secreted from prostate cancer cells under hypoxic condition delivered more signaling factors than normal oxygen condition, including TGF‐β, TNF‐α, and IL‐6, thereby promoting cell invasion by altering the tumor microenvironment [46]. Exosomal TGF‐β and IL‐10 from metastatic lung cancer promoted cell migration under hypoxic conditions [47]. Consistently, hypoxia enhanced exosomal secretion from BCa cells and inhibited T‐cell proliferation by increased exosomal TGF‐β [38]. Cancer cell‐derived sEVs transferred TGF‐β1, leading to a wound‐healing response in myofibroblasts by eliciting Smad3 signaling cascade [48].

The present study demonstrated that sEVs from adriamycin‐resistant MCF‐7 cells carried abundant cytokines than sEVs from the parental cells. Importantly, uptake of the resistant cell‐derived sEVs resulted in enhancing the drug resistance of the sensitive cells. TGF‐β1 was selected to decipher sEV‐mediated intercellular cytokine transferring, which supposedly increases the drug resistance according to KEGG analysis. It has been reported that adriamycin adaptively induces TGF‐β signaling and increases Smad2/3 phosphorylation for survival [30]. Subsequently, the present study elucidated that the activation of TGF‐β1/Smad pathway enhances the adriamycin resistance in the sEV/TGF‐β1 uptake cells via administration of apoptosis and EMT. Consistently, the effect was attenuated after the intervention of the sEV effect by silencing TGF‐β1.

In summary, EVs are thought to be the important transfer vehicles for the intercellular communication and EV‐delivered active molecules remarkably contribute to drug resistance within the tumor microenvironment. Since cytokine is highly associated with drug resistance of BCa, the implication of EV‐mediated cytokine transfer for drug resistance is prospected to be a major concern for BCa treatment. Thus, the insights into EV‐mediated cytokine transfer within the tumor microenvironment are anticipated to provide promising approaches for discovering novel biomarkers and therapeutic targets by capturing EVs.

## 5 Conclusion

Adriamycin‐resistant BCa cell‐derived sEVs conferred the resistant phenotype to sensitive BCa cells via the delivery of TGF‐β1, leading to increased cell growth by inhibiting apoptosis and promoted cell mobility by enhancing EMT.

## Conflict of interest

The authors declare no conflict of interest.

## Author contributions

CT, WS, ZX, and YX conceived and designed the study; CT, WS, ZX, SZ, WH, XW, and ZW developed methodology; CT, ZX, and YX analyzed and interpreted the data; YZ, GZ, YX, and JT provided administrative, technical, or material support; CT, WS, and YX wrote, reviewed, and revised the manuscript; and YX and JT supervised the project.

## Supporting information


**Fig. S1.** Characterization of adriamycin‐resistant MCF‐7 cell line established in this study.
**Fig. S2.** KEGG metastatic pathway analysis based on increased levels of cytokines in A/sEVs vs. S/sEVs.
**Fig. S3.** Quantification of TGF‐β1 expression levels in MCF‐7/Adr vs. parental MCF‐7 cells.
**Fig. S4.** Co‐localization of TGF‐β1 in sEVs.
**Fig. S5.** Establishment of a zebrafish tumor experimental model. Establishment of a zebrafish tumor experimental model.
