# Supplementary material for: Small extracellular vesicles deliver TGF‐β1 and promote adriamycin resistance in breast cancer cells
Source: Mol Oncol. 2021 Feb 24;15(5):1528–42. doi: 10.1002/1878-0261.12908 (PMC8096780; doi:10.1002/1878-0261.12908)
Supplement: Supplementary file 1 — Fig. S1. Characterization of adriamycin‐resistant MCF‐7 cell line established in this study. Fig. S2. KEGG metastatic pathway analysis based on increased levels of cytokines in A/sEVs vs. S/sEVs. Fig. S3. Quantification of TGF‐β1 expression levels in MCF‐7/Adr vs. parental MCF‐7 cells. Fig. S4. Co‐localization of TGF‐β1 in sEVs. Fig. S5. Establishment of a zebrafish tumor experimental model. Establishment of a zebrafish tumor experimental model. [file MOL2-15-1528-s001.docx]

**Supplemental information**


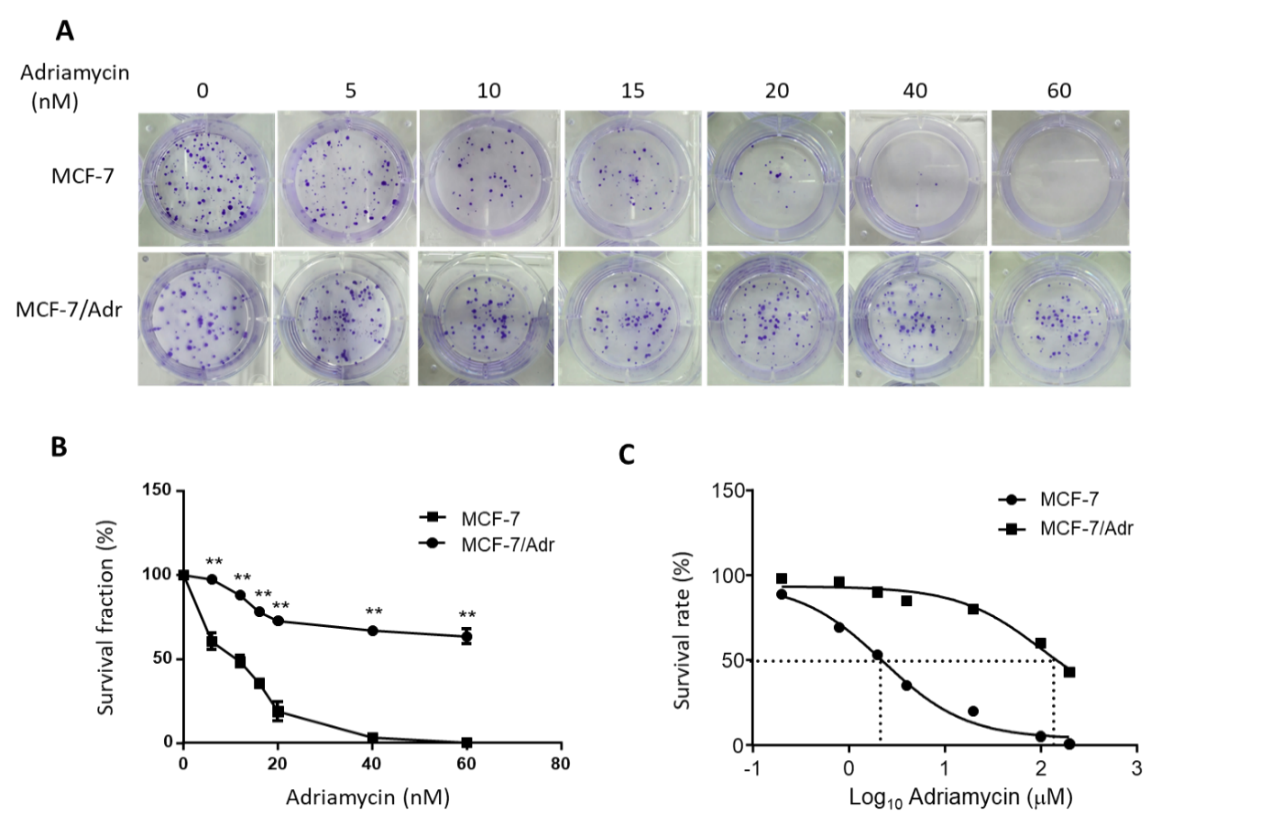


**Figure S1. Characterization of adriamycin-resistant MCF-7 cell line established in this study.** (A) 200 MCF-7/Adr cells vs. parental MCF-7 cells were plated in 6-well plates and then treated with dose-escalated adriamycin as indicated. The treated cells were cultured until the colony formed. (B) The cell colony surviving rates were determined. (C) 1000 cells were plated 96-cell plates and then treated with different doses of adriamycin corresponding to the clognoenic assay. The IC50 value was calculated. The experiments were repeated with more than 3 replicates. The data was presented as the mean ± standard deviation. **(*P*< 0.01) presents the significances between the two groups as indicated.


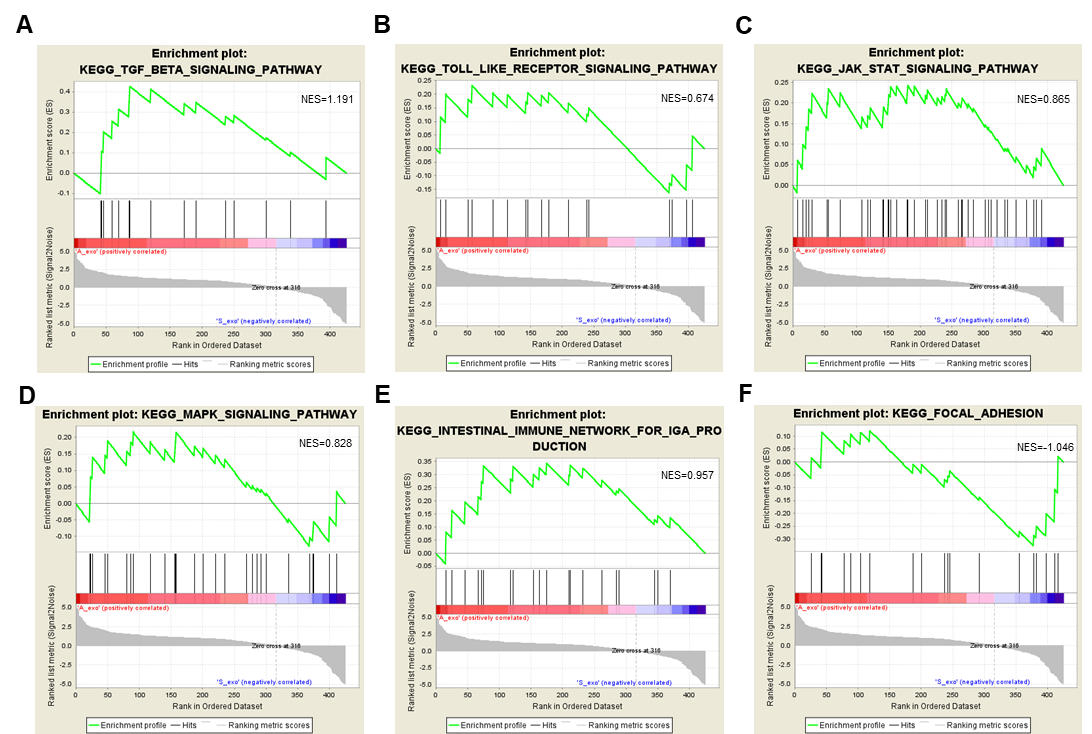


**Figure S2. KEGG metastatic pathway analysis based on increased levels of cytokines in A/sEVs vs. S/sEVs.** (A-F) Increased abundances of cytokines in A/sEVs vs. S/sEVs were determined by cytokine antibody array. KEGG pathway analysis enriched several metastatic signaling pathways involved as indicated.

**
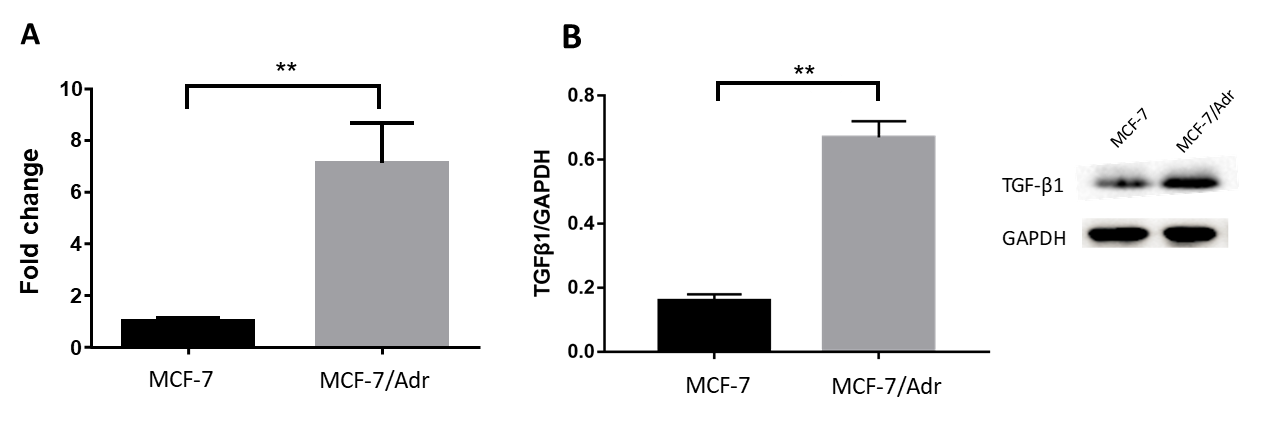
**

**Figure S3. Quantification of TGF-β1 expression levels in MCF-7/Adr vs. parental MCF-7 cells.** (A) mRNA levels of TGF-β1 in MCF-7/Adr vs. MCF7 cells were measured by RT-qPCR. (B) Accordingly, protein levels of TGF-β1in the two cell lines were quantified by western blots. The experiments were repeated with more than 3 replicates. The data was presented as the mean ± standard deviation. **(*P*< 0.01) presents the significances between the two groups as indicated.

**
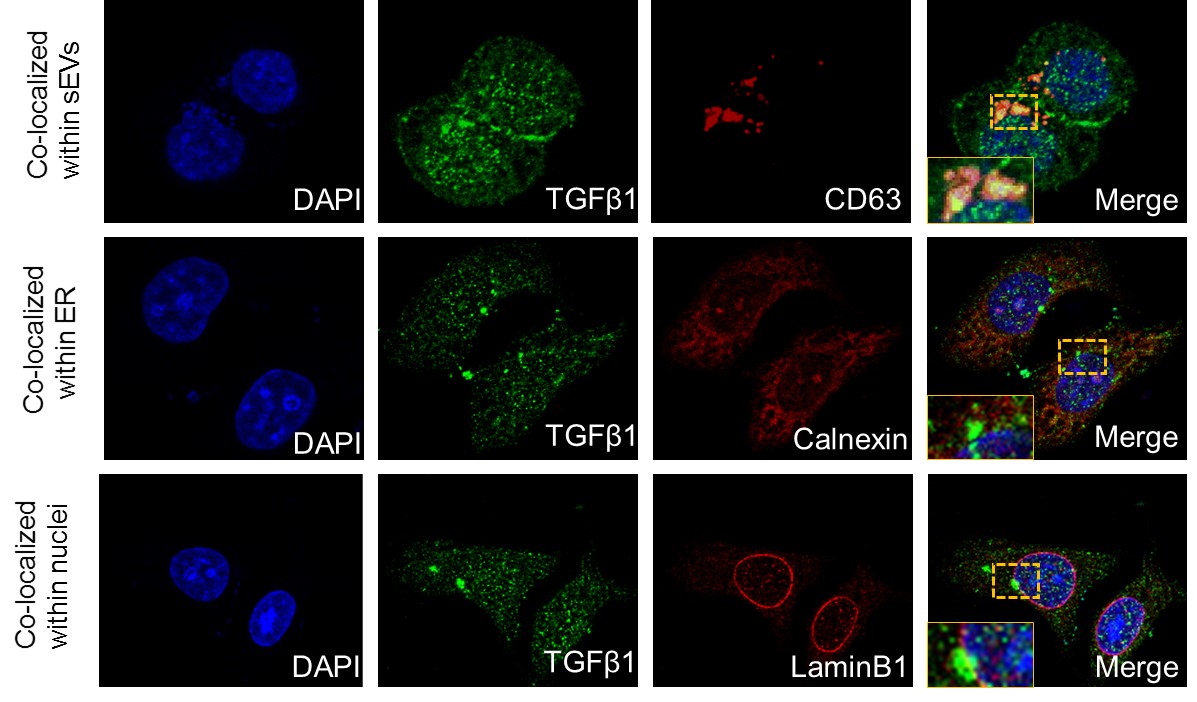
**

**Figure S4. Co-localization of TGF-β1 in sEVs.** After cell-A/sEVs incubation, the cells were stained with DAPI (blue), TGF-β1 (green), CD63/Calnexcin/LaminB1 (red). Color merges indicate the co-location of TGF-β1 with the three membrane proteins, which enlarged in the left bottom of the merged images.

**Figure S5. Establishment of a zebrafish tumor experimental model.** (A) MCF-7/Adr cells and parental MCF-7 cells were labeled with PKH26 and injected into zebrafish embryos. Adriamycin was added into zebrafish nutrient solution to inhibit tumor growth. The tumors were imaged using a fluorescent microscope. (B) Tumor volumes were measured and plotted. 10-15 fishes in each experimental group and the data was presented as the mean ± standard deviation. *(*P*<0.05) and **(*P*< 0.01) present the significances between the two groups as indicated.
